# Differential Frequencies of Intermediate Monocyte Subsets Among Individuals Infected With Drug-Sensitive or Drug-Resistant *Mycobacterium tuberculosis*

**DOI:** 10.3389/fimmu.2022.892701

**Published:** 2022-07-15

**Authors:** Pavithra Sampath, Alangudi Palaniappan Natarajan, Kadar Moideen, Gokul Raj Kathamuthu, Syed Hissar, Madhavan Dhanapal, Lavanya Jayabal, Paranchi Murugesan Ramesh, Srikanth Prasad Tripathy, Uma Devi Ranganathan, Subash Babu, Ramalingam Bethunaickan

**Affiliations:** ^1^ Department of Immunology, National Institute for Research in Tuberculosis (NIRT), Chennai, India; ^2^ Department of Clinical Research, National Institute for Research in Tuberculosis (NIRT), Chennai, India; ^3^ Indian Council of Medical Research-National Institute for Research in Tuberculosis (ICMR-NIRT)-International Center for Excellence in Research, Chennai, India; ^4^ Corporation of Chennai, Chennai, India; ^5^ Government Hospital for Thoracic Medicine (GHTM), Chennai, India; ^6^ Indian Council of Medical Research-National Institute for Research in Tuberculosis (ICMR-NIRT), Chennai, India

**Keywords:** monocytes and dendritic cell subsets, drug-resistant TB (DR-TB), immunophenotyping, uniform manifold approximation and projection (UMAP), TB immune responses, monocyte to lymphocyte ratio, neutrophil to lymphocyte ratio

## Abstract

The rampant increase in drug-resistant tuberculosis (TB) remains a major challenge not only for treatment management but also for diagnosis, as well as drug design and development. Drug-resistant mycobacteria affect the quality of life owing to the delayed diagnosis and require prolonged treatment with multiple and toxic drugs. The phenotypic modulations defining the immune status of an individual during tuberculosis are well established. The present study aims to explore the phenotypic changes of monocytes & dendritic cells (DC) as well as their subsets across the TB disease spectrum, from latency to drug-sensitive TB (DS-TB) and drug-resistant TB (DR-TB) using traditional immunophenotypic analysis and by uniform manifold approximation and projection (UMAP) analysis. Our results demonstrate changes in frequencies of monocytes (classical, CD14^++^CD16^-^, intermediate, CD14^++^CD16^+^ and non-classical, CD14^+/-^CD16^++^) and dendritic cells (DC) (HLA-DR^+^CD11c^+^ myeloid DCs, cross-presenting HLA-DR^+^CD14^-^CD141^+^ myeloid DCs and HLA-DR^+^CD14^-^CD16^-^CD11c^-^CD123^+^ plasmacytoid DCs) together with elevated Monocyte to Lymphocyte ratios (MLR)/Neutrophil to Lymphocyte ratios (NLR) and alteration of cytokine levels between DS-TB and DR-TB groups. UMAP analysis revealed significant differential expression of CD14^+^, CD16^+^, CD86^+^ and CD64^+^ on monocytes and CD123^+^ on DCs by the DR-TB group. Thus, our study reveals differential monocyte and DC subset frequencies among the various TB disease groups towards modulating the immune responses and will be helpful to understand the pathogenicity driven by *Mycobacterium tuberculosis.*

## Introduction

According to the global tuberculosis (TB) report, in 2021, about 10 million people developed TB which resulted in 1.3 million deaths. Among these, India contributes about 2.64 million to TB incidence. The end TB strategy emphasizes efforts directed at the development and identification of new vaccines, diagnostic biomarkers and therapeutic modalities. The emergence of rifampicin resistance and multi-drug resistance is a major threat to humans and requires expensive drugs and prolonged treatment (1). Identification and treatment of latent TB (LTB) infection is another challenge in TB research as 5 to 10% of latently infected people are at risk of progressing to active TB; this risk depends on host immunity that determines the fate of mycobacterium.

Analyzing immune cells through phenotypic and functional characterization becomes crucial as they may represent the *in-vivo* disease state and provide an understanding of the unresolved immunopathology of the infection (2, 3). Among various immune responses, monocytes transition to macrophages that are frontline defenders, providing innate defense against *Mycobacterium tuberculosis* (MTB) infection by controlling their replication (4–7). Peripheral blood monocytes broadly divide into three major subsets, classical, CD14^++^CD16^-^, intermediate, CD14^++^CD16^+^ and non-classical, CD14^+/-^CD16^++^ (8–10) with diverse functions (11, 12). During TB infection, CD16 monocytes exhibit perturbation and are associated with the disease severity (4, 13). Monocyte subsets also modulate their cytokine production under infectious and non-infectious conditions (4, 14). Numerous studies spotlighted circulating monocytes and monocyte to lymphocyte ratio (MLR) are better discriminators of latent infection from active TB and describe the immune efficiency of an individual, TB disease progression and the treatment outcome (15–21).

Monocytes can also fuel the adaptive immune response by differentiating into dendritic cells (DC) (8). DCs respond to mycobacteria by sensing their pathogen-associated molecular patterns (PAMP) through toll-like receptors (TLR) and migrate to and from lung tissue with altered subset phenotype (22–25). Mycobacteria hinder the differentiation ability of monocytes to DC (26) and their antigen presentation ability (27). The blood DC phenotype may be linked with treatment outcome, prolonged/complicated TB and the lymphocyte response to infection (28).

In this study, we aimed to explore the blood phenotype of individuals with the spectrum of TB infection from latency to drug-sensitive and drug resistance exclusive of co-morbidities. We demonstrated phenotypic differences of monocytes and DC subsets across the study groups and highlighted the implication of MLR as the potent marker for TB disease. In addition, we also performed the uniform manifold approximation and projection (UMAP) analysis to understand the differential expression of blood (monocytes and DC) phenotypes. On UMAP analysis, we observe that DR-TB has a different expression of monocyte and DC markers compared to other study groups.

## Materials and Methods

### Study Subject Recruitment and Clinical Parameters

This study was approved by the Institutional Ethics Committee (IEC) of the National Institute for Research in Tuberculosis (NIRT, IEC 2015022), Chennai, India. Blood samples were collected from 160 study participants in four groups inclusive of healthy volunteers (n=40), latently infected individuals (n=40), drug-sensitive pulmonary TB patients (n=40) and multi drug-resistant patients (n=40) (**Table 1**). Individuals who are asymptomatic for TB with no past TB history, normal chest X-ray and found negative for Interferon Gamma Release Assay (IGRA) are categorized as healthy controls (HC) (Group-1). Individuals who are asymptomatic for TB with no past TB history, normal chest X-ray and found positive for IGRA are categorized as latent TB (LTB) (Group-2). Patients diagnosed with pulmonary TB who are sensitive to TB first-line drugs with typical clinical and/or radiological presentation, sputum CBNAAT/smear and/or culture positivity for MTB are categorized as DS-TB (Group-3). Patients identified as multi-drug resistant by drug sensitivity test for first-line anti-TB treatment drugs, such as Isoniazid and Rifampicin are categorized as DR-TB (Group-4). All study participants were recruited from in and around Chennai with the age ranges between 18 years and below 55 years who had given written informed consent. Heparinized blood was collected before the commencement of anti-TB treatment (ATT). Individuals with extra-pulmonary TB, viral infections (HIV, HBV & HCV), diabetes, autoimmune conditions, psychiatric illness, those receiving immunosuppressant drugs and those undergoing anti-tuberculosis treatment were excluded from the study.

**Table 1:** Demographics of the study population.

Groups	HC	LTB	DS-TB	DR-TB
No. of subjects recruited	40	40	40	40
Gender (Male/Female)	20/20	18/22	28/12	32/8
Age (Median, IQR)	27 (12)	29 (14.5)	40 (19.25)	36 (24)

### Hematology Profiling

A complete differential blood counting was performed using a hematology analyzer (Mindray BC-5150). The ratio of monocytes to lymphocytes (MLR) and neutrophils to lymphocytes (NLR) was determined and compared among the four different study groups.

### Cell Surface Marker Immunostaining

All the reagents and antibodies used in the study were procured from BD Biosciences, BD Pharmingen and Lonza India PVT LTD. Anti-human monoclonal antibodies used are CD3 FITC (clone: HIT3a, BD), CD19 FITC (clone: HIB19, BD), CD56 FITC (clone: B159, BD), CD2 FITC (clone: RPA-2.10, BD), HLA-DR PE Cy7 (clone: G46-6, BD), CD14 Per CP (clone: MϕP9, BD), CD16 Pacific Blue (clone: 3G8, BD), CD64 PE (clone: 10.1, BD), CD86 APC (clone: 2331(FUN-1), BD), CD141 PE (clone: 1A4, BD), CD123 APC (clone: 7G3, BD) and CD11c Alexa Flour 700 (clone: B-ly6, BD). Human whole blood (250 µl) was labelled with an antibody cocktail specifically intended for characterizing monocyte and DC phenotypes and FACS tubes were incubated for 30 minutes at 4°C. Following antibody addition, erythrocyte lysis was performed by adding BD FACS lysing solution (BD Biosciences) and incubated for 10-20 minutes at room temperature. After lysis, cells were subjected to wash twice with 2ml of 1X PBS and pelleted upon centrifugation at 1200 rpm for 10 minutes. The final pellet was fixed with 2% paraformaldehyde solution and stored at 4°C until acquisition. Flow cytometry analysis was performed on eight colour fluorescence FACS Canto II (BD, USA) flow cytometer instrument using FACS DIVA software (version 6.0). One million gated events were acquired and the data were analyzed by FlowJo software (version 10.0.1) to determine the frequency of monocyte and DC subsets. The monocyte population was identified as lineage negative (CD2^-^CD3^-^CD19^-^CD56^-^), HLA-DR^+^ and HLA-DR^+^CD16^+^ cells. Subsets of monocytes were classified as classical, CD14^++^CD16^-^, intermediate, CD14^++^CD16^+^ and non-classical, CD14^+/-^CD16^+^ monocytes. DCs were identified as lineage negative (CD3^-^CD19^-^CD56^-^), HLA-DR^+^, CD14^-^ and CD16^-^ cells. DCs are gated as HLA-DR^+^CD11c^+^ myeloid DCs (mDC), cross-presenting HLA-DR^+^CD14^-^CD141^+^ mDCs and HLA-DR^+^CD14^-^CD16^-^CD11c^-^CD123^+^ plasmacytoid DCs (pDC).

### Dimensionality Reduction by UMAP

UMAP (nonlinear, dimension reduction) analysis was performed (n=40, 10 individuals in each [DR-TB, DS-TB, LTB, HC] group) with the help of FlowJo plugins available in FlowJo™ 3 (version 10, [TreeStar, Ashland, OR]) software. We down-sampled 10000 cells (from each sample) of HLA-DR^+^, CD16^+^ monocytes and HLA-DR^+^ DCs for the concatenation and performed the UMAP analysis using default parameters. The significance of the mean fluorescent intensity (MFI) values on UMAP analysis between the study groups was determined using the chi-square test.

### Circulating Levels of Cytokines

Plasma from all the blood samples was separated and stored at -20°C. Plasma samples were thawed and circulating cytokines TNFα, IL-6, IL-2, IL-1β and IL-10 were measured by ELISA kit (DuoSet) from R&D systems, according to the protocol as per the manufacturer’s instructions. The data were analyzed by SoftMax Pro software and the cytokine concentration (picograms/milliliter) was interpolated using standards plotted in the curve fit.

### Statistical Analysis

All the data were analyzed by non-parametric Kruskal Wallis test using graph pad prism software (Version 8). p-value less than 0.05 is considered statistically significant and the level of significance is denoted as * [p<0.01(*); p<0.001(**); and p<0.0001(***)].

## Results

### Elevated Hematological Features (Monocyte Numbers, MLR and NLR) and Their Implication to for Clinical Severity

**Figure 1:**
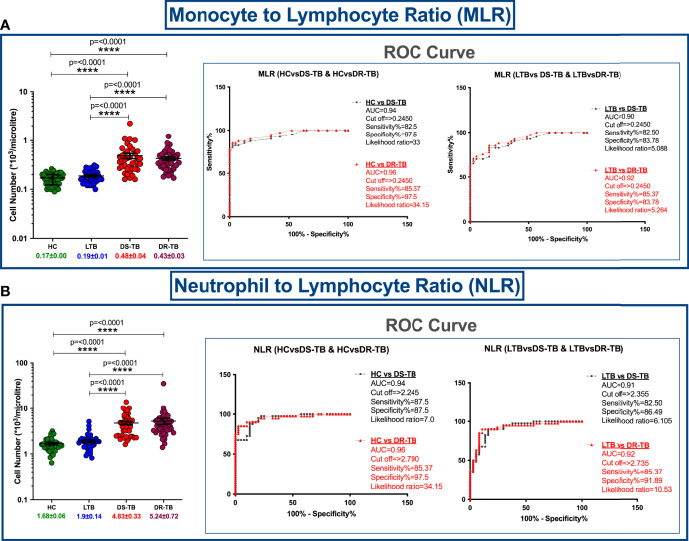
Cell number distribution of monocyte to lymphocyte ratio (MLR) **(A)** and neutrophil (NLR) to lymphocyte ratio **(B)** among four groups with ROC curves. The significance level with their p values are mentioned in the graph. Highly significant ones are mentioned as **** where p=<0.0001.

Hematological features such as monocyte numbers, MLR and NLR are represented in **Figure 1**. **Figure 1A, B** show a significant elevation of MLR and NLR (p<0.0001) along with specificity, sensitivity, likelihood ratio and area under curve (AUC) value (>0.9) among DS-TB and DR-TB groups compared to HC and LTB groups. The MLR cutoff >0.245 for HC vs DS-TB (sensitivity 82.5%, specificity 97.5% and likelihood ratio 33) and HC vs DR-TB (sensitivity 85.37%, specificity 97.5% and likelihood ratio 34.15) and NLR cutoff >2.790 for HC vs DR-TB (sensitivity 85.37%, specificity 97.5% and likelihood ratio 34.15) yielded maximum likelihood ratio and better discriminating specificity.

### Altered Monocyte Subsets Frequencies Associated With DS-TB and DR-TB Groups

**Figure 2:**
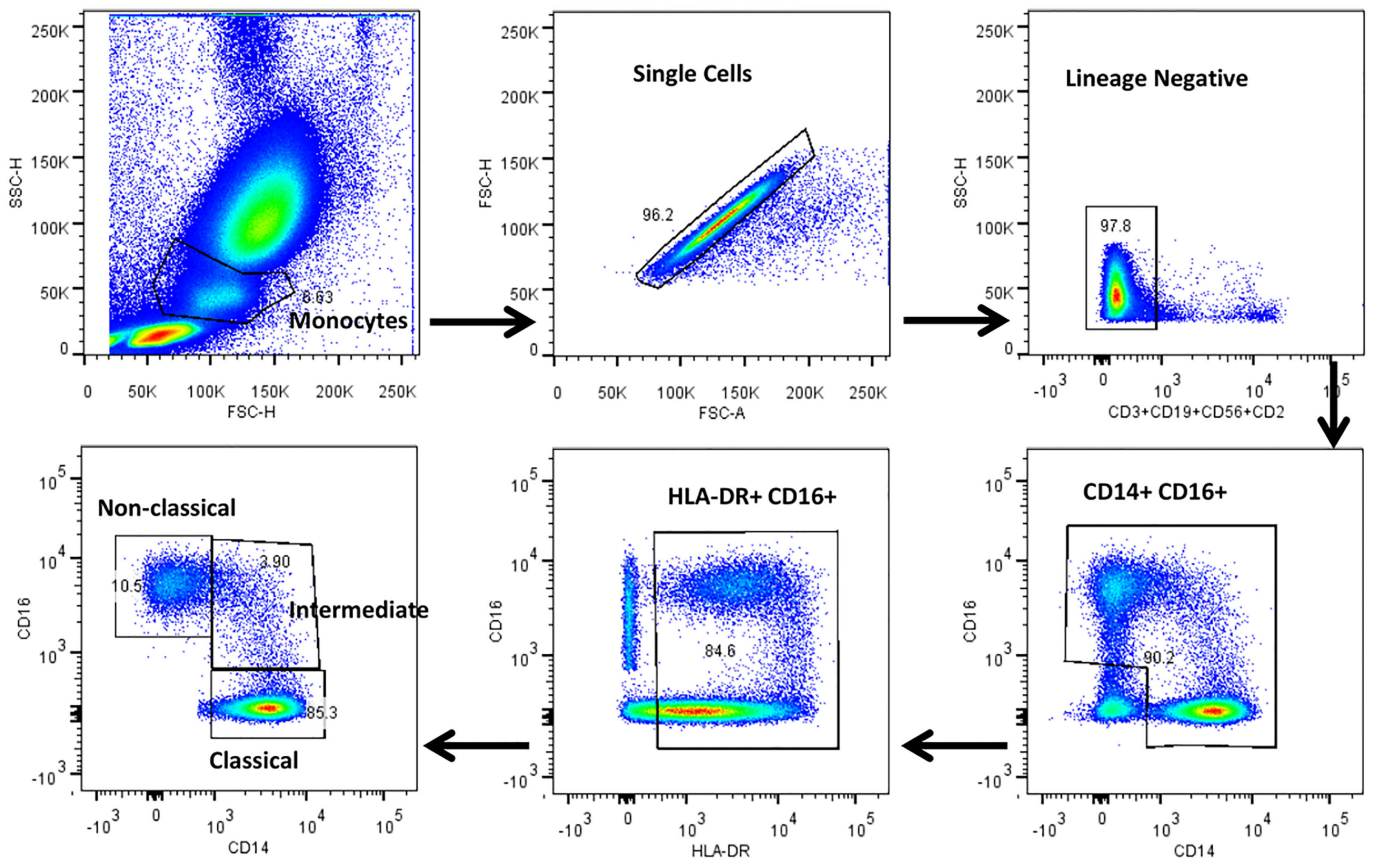
Representative gating strategy for monocytes and their subsets. Monocyte populations were gated on whole blood cell plot by Forward (FSC-A) and Side scatter (SSC-A) based on their size and granularity. Singlets were discriminated against doublets by FSC (A) vs FSC (H). Dump gate were used to select lineage negative cells by excluding T cells, B cells and NK cells by CD2, CD3, CD19 and CD56 markers. Total monocytes were gated using CD14 and CD16 surface expression. HLA-DR+ and CD16+ cells were gated to obtain the pure monocyte population which were further gated using CD14 and CD16 surface markers to distinguish classical (HLADR^+^CD14^++^CD16^-^), intermediate (HLADR^+^CD14^++^CD16^+^) and non-classical (HLADR^+^CD14^+/-^CD16^+^) monocytes.

**Figure 3:**
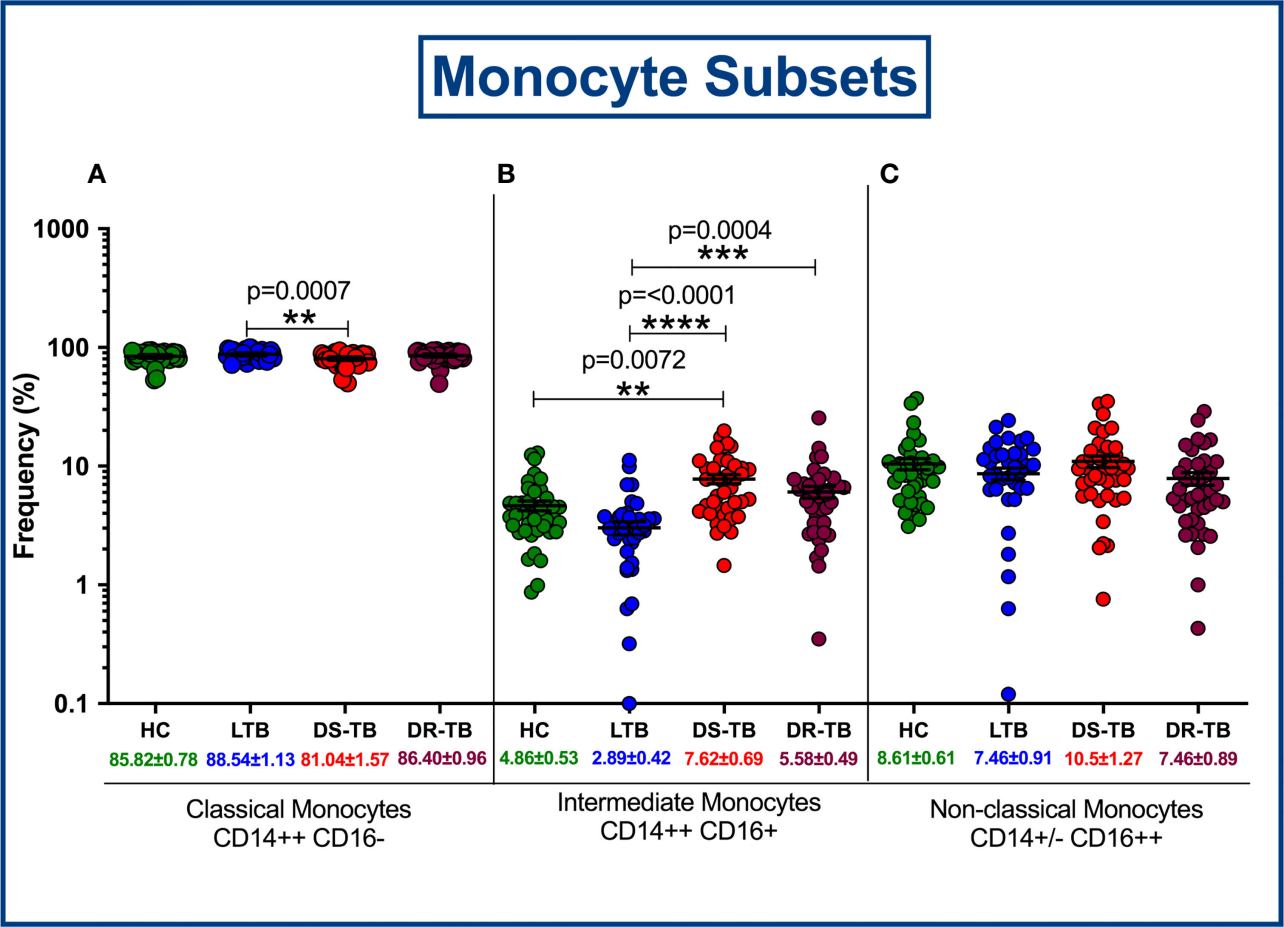
Frequency distribution of monocyte subsets; Classical monocytes **(A)**, Intermediate monocytes **(B)** and Non-classical monocytes **(C)**.

The gating strategy for monocytes and their subsets were defined based on cell surface expression of HLA-DR, CD14 and CD16 markers (**Figure 2**). We displayed the modulated frequencies of monocyte subsets among HC, LTB, DS-TB and DR-TB groups (**Figure 3**). We show significant differences in the DS-TB group with a diminished classical monocyte subset (**Figure 3A**) compared to the LTB group. Similarly, we show elevated intermediate monocytes in DS-TB compared to LTB and HCs (**Figure 3B**). Additionally, we delineate the elevated intermediate subset in DR-TB compared to the LTB group (**Figure 3B**). Finally, we did not observe any significant difference in the non-classical subset among the study groups (**Figure 3C**).

### Altered DC Frequencies Associated With DS-TB and DR-TB Groups

**Figure 4:**
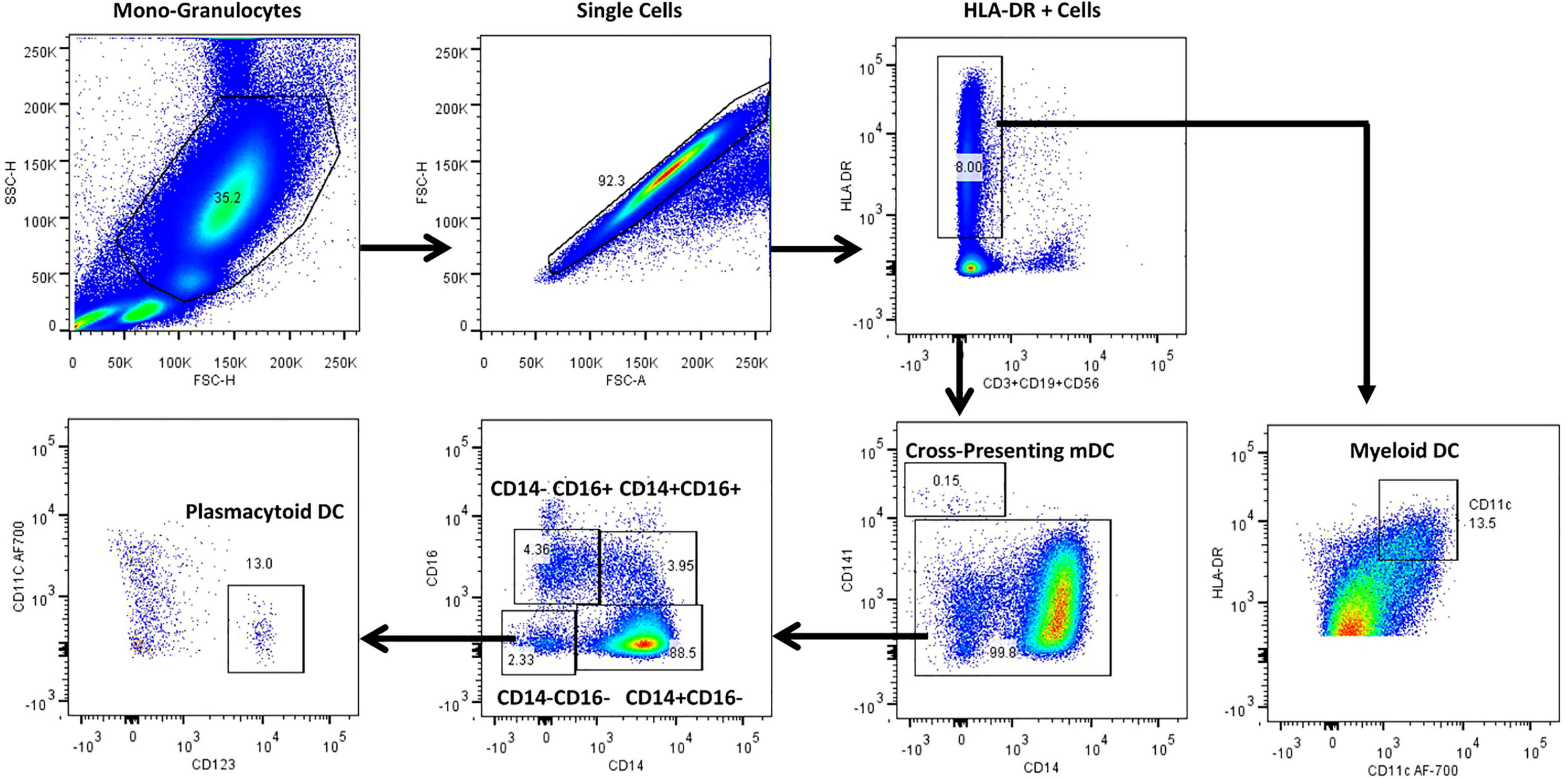
Representative gating strategy for dendritic cells and their subsets. Monocyte and granulocyte population were gated on whole blood cell plot excluding lymphocytes by Forward (FSC-A) and Side scatter (SSC-A) based on its size and granularity. Singlets were discriminated from doublets by FSC (A) vs FSC (H). Lineage negative cells were identified with the help of dump gate by excluding T cells, B cells and NK cells using CD3, CD19 and CD56 markers. HLA-DR+ were gated to obtain HLA-DR^+^CD11c^+^ myeloid DCs (mDC), cross-presenting HLA-DR^+^CD14^-^CD141^+^ myeloid DCs and HLA-DR^+^CD14^-^CD16^-^CD11c^-^CD123^+^plasmacytoid DCs (pDC).

**Figure 5:**
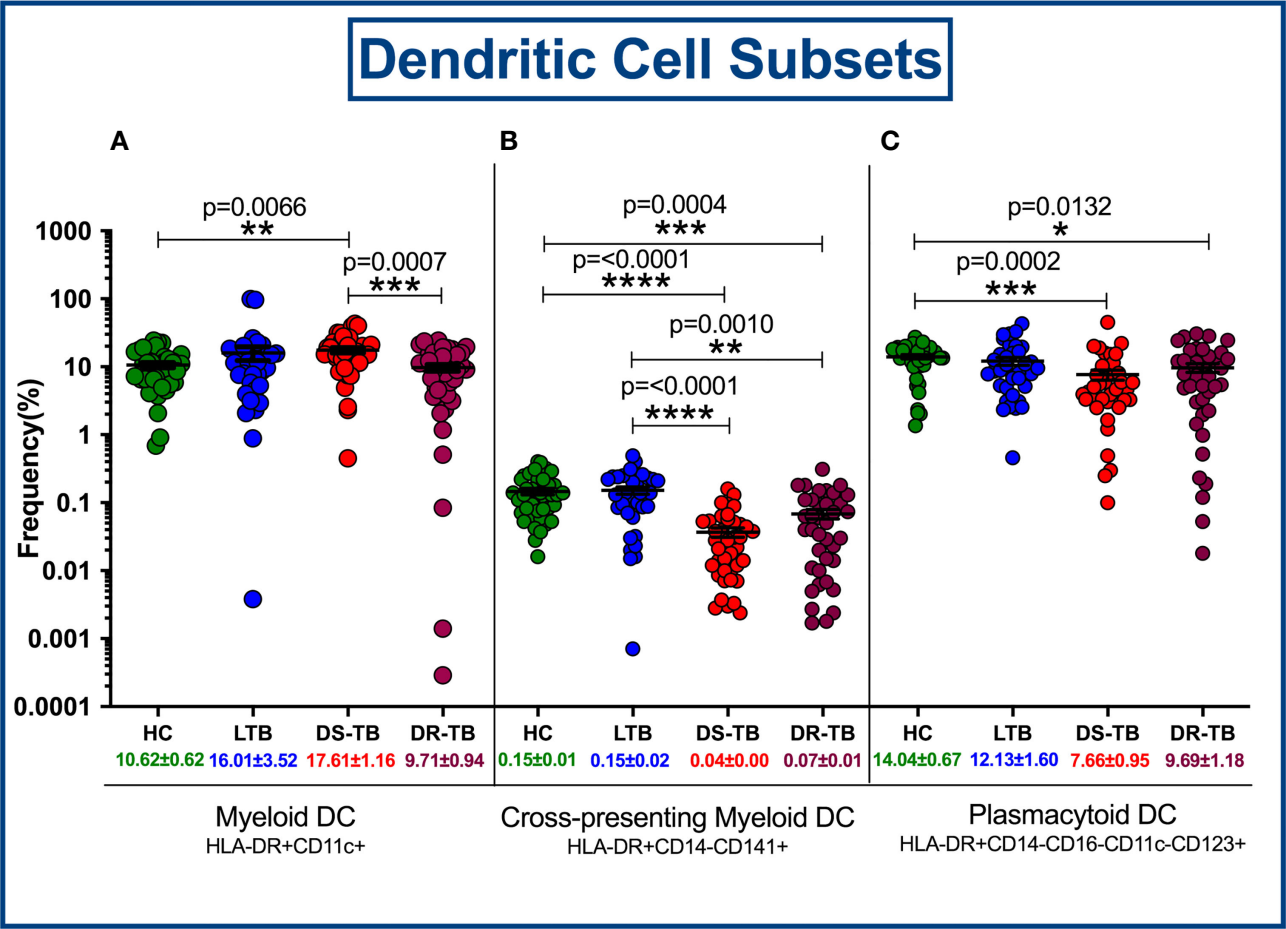
Frequency distribution of dendritic cell subsets; HLA-DR^+^CD11c^+^ myeloid DCs **(A)**, Cross-presenting myeloid DCs **(B)** and Plasmacytoid DCs **(C)** among four groups. p value less than 0.05 is considered as statistically significant and the level of significance denoted as * [p<0.01(*); p<0.001(**); and p<0.0001(***)]. The significance level with their p values are mentioned in the graph. Highly significant ones are mentioned as **** where p=<0.0001.

**Figure 4** represents the gating strategy for DC and their subsets. They were defined based on cell surface expression of HLA-DR, CD11c, CD123, CD141, CD14 and CD16 markers. We displayed the frequencies of DC subsets among HC, LTB, DS-TB and DR-TB groups (**Figure 5**). We delineate that HLA-DR^+^CD11c^+^ mDC frequencies were significantly elevated in DS-TB compared to HCs and DR-TB group but not with the LTB group (**Figure 5A**). We show both DS-TB and DR-TB groups associated with significantly diminished frequencies of cross-presenting HLA-DR^+^CD14^-^CD141^+^ mDCs (**Figure 5B**) and HLA-DR^+^CD14^-^CD16^-^CD11c^-^CD123^+^ pDCs (**Figure 5C**) compared to HC and/or LTB groups.

### High Dimensionality Reduction Analysis of Monocytes Subsets by UMAP Analysis

**Figure 6:**
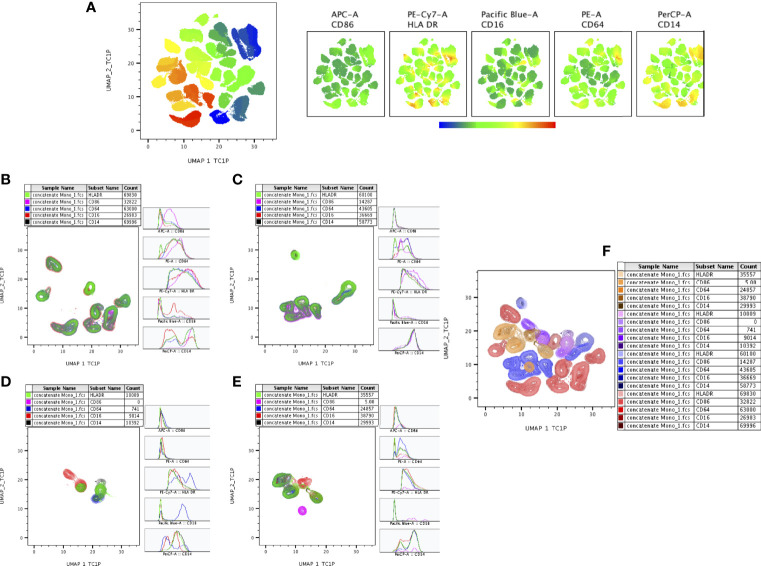
UMAP analysis of monocyte subsets. CD16^+^HLADR^+^ (7,000 cells) were down sampled from monocytes and performed **(A)** unsupervised (UMAP (UMAP_1_TC1P vs UMAP_2_TC1P) clusters (ungated) and expression of individual monocytes (CD86, HLADR, CD14, CD16, CD64) markers between the study (DR-TB, DS-TB, LTB, HC) population. **(B–E)** represents the DR-TB, DS-TB, LTB, HC group expression of monocyte marker (islands) subsets using contour plots. **(F)** shows merged contour plot expression of monocyte subsets among the study individuals.

The expression of monocyte (CD14, CD16, CD64, CD86, HLA-DR), markers of DR-TB, DS-TB, LTB and HC individuals are represented in **Supplementary Figure 1**. **Figure 6A** represents ungated monocyte markers expression of DR-TB, DS-TB, LTB and HC individuals on UMAP plot. As shown in **Figure 6B–E**, DR-TB group was associated with significant differential expression of CD14, CD16, CD64, CD86 and HLA-DR markers compared to DS-TB, LTB and HC groups. Finally, we also depicted the merged expression of CD14, CD16, HLA-DR, CD64 and CD86 markers between the four study groups (**Figure 6F**).

### High Dimensionality Reduction Analysis of DC Subsets by UMAP Analysis

**Figure 7:**
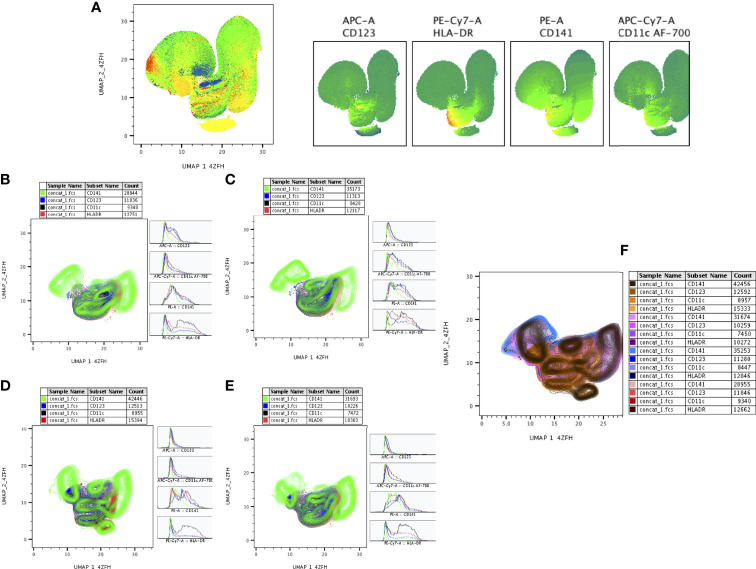
UMAP analysis of DC subsets. HLADR^+^ (10,000 cells) were down sampled for monocytes/granulocytes and performed **(A)** unsupervised (UMAP (UMAP_1_4ZFH vs UMAP_2_4ZFH) clusters (ungated) and expression of individual DC (CD123, HLADR, CD141, CD11c) markers between the study (DR-TB, DS-TB, LTB, HC) population. **(B–E)** shows the DR-TB, DS-TB, LTB and HC group expression of DC marker (islands) subsets using contour plots. **(F)** shows merged contour plot expression of DC subsets among the study individuals.

The expression of DC (CD11c, CD123, CD141, HLA-DR) markers of DR-TB, DS-TB, LTB and HC individuals are represented in **Supplementary Figure 2**. We represented (**Figure 7A**) overall ungated DC markers expression in DR-TB, DS-TB, LTB and HC individuals on UMAP plot. As shown in **Figures 7B–E**, DR-TB group associated with significantly different expression of CD141^+^ marker compared to DS-TB, LTB and HC individuals. In contrast, three different (DR-TB, DS-TB and LTB) diseased groups have significant diverse expression of CD11c^+^, HLA-DR^+^ and CD123^+^ markers compared to HC individuals (**Figures 7B–E**). We also displayed the merged expression of CD11c, HLA-DR, CD123 and CD141 markers between the study individuals (**Figure 7**).

### Circulating Levels of Pro/Anti-Inflammatory Cytokines

**Figure 8:**
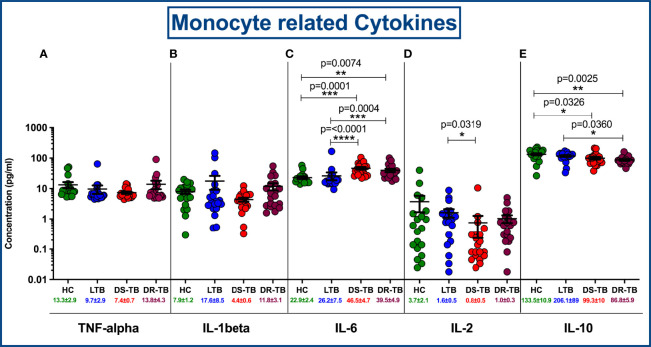
Comparison of mean concentration of circulating pro and anti-inflammatory cytokines, TNF-α **(A)**, IL-1β **(B)**, IL-6 **(C)**, IL-2 **(D)** and IL-10 **(E)** among four groups. p value less than 0.05 is considered as statistically significant and the level of significance denoted as * [p<0.01(*); p<0.001(**); and p<0.0001(***)]. The significance level with their p values are mentioned in the graph. Highly significant ones are mentioned as **** where p=<0.0001.

The circulating levels of pro (TNFα, IL-1β, IL-6 and IL-2) and anti-inflammatory (IL-10) cytokines were represented in **Figures 8A–E**. We show the marked differences in DS-TB and DR-TB groups with elevated pro-inflammatory IL-6 (**Figure 8C**) and decreased anti-inflammatory IL-10 (**Figure 8E**) levels compared to HC and LTB groups. Similarly, IL-2 levels was signifcantly reduced in DS-TB compared to LTB alone (**Figure 8D**). Finally, we did not observe any notable differences among other pro-inflammatory cytokines (TNFα and IL-1β) between the study groups.

## Discussion

The growing evidence of the altered phenotypic profile of immune cells during TB (4, 13, 28, 29) intrigued us towards exploring the monocyte and DC phenotypes across the TB disease spectrum from latency to drug sensitivity and drug resistance conditions. Through this, we obtained a holistic picture of condition-specific changes at one-time point before treatment initiation. The predefined changes with respect to altered monocyte (decreased classical, increased intermediate) (4, 30) and DC subsets (decreased cross-presenting myeloid and plasmacytoid DC) (28, 31), elevated MLR/NLR (15–19) and altered cytokine profile (increased IL-6) (32, 33) in TB groups compared to the healthy group are in conjunction with published studies.

During MTB infection, there is a shift in the subset distribution of monocyte and DC thereby they are successful in evading the host innate and adaptive immune responses. Upon MTB stimuli, the classical monocyte subset migrates faster to the infection site and differentiates into intermediate and non-classical subsets by acquiring CD16 expression. This might attribute to the possible drop in the CD14 positive classical subset and the rise in the CD16 positive intermediate subset (but not with the non-classical subset in our data) during TB disease. Accumulation of CD16 positive subset in periphery attributes inflammation, MTB dissemination and severity as these cells are more permissive for MTB growth and replication (14). In addition, the expanded CD16 monocytes are associated with impaired dendritic cell differentiation (26) thus leading to fall in the DC population and linked to hindered antigen presentation with inefficient priming of CD4 T cells and IFN mediated killing (31). Also, the accumulation of DCs in tuberculous granuloma could be the other potential reason for their loss in the peripheral blood (34). Thus, the adaptive immunity is disoriented as the total lymphocyte count is reduced which is well observed with the elevated MLR/NLR defining the TB progression and severity (15–19).

Perplexed immune cells alter their balanced secretion of pro- and anti-inflammatory cytokines. Bifunctional IL-6 and their increased levels in the DS-TB and DR-TB groups at baseline are often associated with smear grade, bacterial load, radiological severity, and unfavourable outcomes (32, 33). This pleiotropic IL-6 cytokine could inhibit the production of TNFβ and IL-1β (35) and therefore their higher levels reduced IL-1β levels in diseased groups though statistically not significant. The expression of other pro-inflammatory cytokines (TNF-α and IL-2) are different from the existing studies (36–39). The reduced IL-10 levels in TB groups are quite contrary to the reported studies (40–42) and were associated with greater pathogen clearance; however, loss of immunity was observed during re-infection. This observation clearly explains reduced IL-10 levels are a key factor in preserving the effector memory populations through an unknown mechanism (43). The above observations from our study and their differences to the existing studies might be limited due to the smaller sample size in the cytokine analysis.

The observed differential frequencies of monocyte and DC subsets in the DR-TB group are trivial and statistically not significant when compared to DS-TB. However, an enhanced disease-mediated activation within intermediate monocyte subsets of DR-TB was noticed through higher mean fluorescence intensity (MFI) values of HLA-DR and CD86 markers (data not shown). In addition, with the high-dimensional approach, the DR-TB group stands out with distinctly different expressions of monocyte (CD16, HLA-DR, CD64 and CD86) and DC (CD123) markers. This indicates their inherent inability in activating the balanced innate and adaptive immunity to fight against TB infection. The possible reason behind these minimal differences may be due to (i) true reflection of drug resistance condition as the intrinsic component for resistance is pathogen-attributed and not host-specific, or (ii) drug-imposed normalcy (similar to HC group expression) as most of the samples are drug acquired resistance, and or (iii) smaller sample size and cross-sectional study design. From our observation, it was much evident that better discrimination between DR-TB and DS-TB was not possible with immunophenotyping of monocyte and DC subsets along with circulating cytokines. This can be resolved with further high-throughput approaches such as single-cell transcriptomics, miRNA regulation, and epigenetic modulation. These approaches can reveal the pathophysiological behavior of these subsets and their dysregulated mechanisms that would be helpful to formulate personalized host-directed therapeutic approaches. Multiplex assays on cell-specific immunological analytes such as cytokines, chemokines, lipid mediators, eicosanoids, and exosomes would provide a better understanding of disease-mediated inflammation and dissemination. To move forward, a longitudinal and multifactorial approach with different samples (bronchial lavage fluid and animal lung tissues) and subgroups (drug acquired and primary resistance) in addition to blood may provide information on lung phenomena with respect to disease risk, remission, and relapse. Exploring further on intermediate monocyte subset and DC subsets both phenotypically and functionally can be helpful to understand the host immune system among DR-TB patients.

## Data Availability Statement

The data supporting the conclusions of this article will be made available by the corresponding author, upon request.

## Ethics Statement

The studies involving human participants were reviewed and approved by Indian Council of Medical Research ICMR-NIRT, Institutional Ethics Commitee. The patients/participants provided their written informed consent to participate in this study.

## Author Contributions

Designed research studies: PS, UR and RB. Conducted experiments, data analysis and interpretation: PS, KM, GK, MD, RB. Contributed towards clinical and instrumental resources; NAP, SH, LJ, RPM and SB. Wrote the manuscript; PS, GK and RB. Manuscript review and editing: SPT, UR, SB and RB. All authors read and approved the final manuscript.

## Funding

This work was funded by the DBT Ramalingaswami Fellowship (Grant Number BT/RLF/Re-entry/38/2013, dated:24.07.2015), Department of Biotechnology, Ministry of Science and Technology, Govt. of India. PS work has been supported by the DST INSPIRE fellowship.

## Conflict of Interest

The authors declare that the research was conducted in the absence of any commercial or financial relationships that could be construed as a potential conflict of interest.

## Publisher’s Note

All claims expressed in this article are solely those of the authors and do not necessarily represent those of their affiliated organizations, or those of the publisher, the editors and the reviewers. Any product that may be evaluated in this article, or claim that may be made by its manufacturer, is not guaranteed or endorsed by the publisher.

## Supplementary Material

Supplementary Figure 1 Individual expression of monocyte (CD14. CD16, HLADR, CD64 and CD86) markers on UMAP between the study populations.

Supplementary Figure 2 Individual expression of DC (CD11c, CD123, CD141 and HLADR) markers on UMAP between the study populations.
