# Supplementary figures and images for: Differential Frequencies of Intermediate Monocyte Subsets Among Individuals Infected With Drug-Sensitive or Drug-Resistant Mycobacterium tuberculosis
Source: Front Immunol. 2022 Jul 15;13:892701. doi: 10.3389/fimmu.2022.892701 (PMC9336531; doi:10.3389/fimmu.2022.892701)

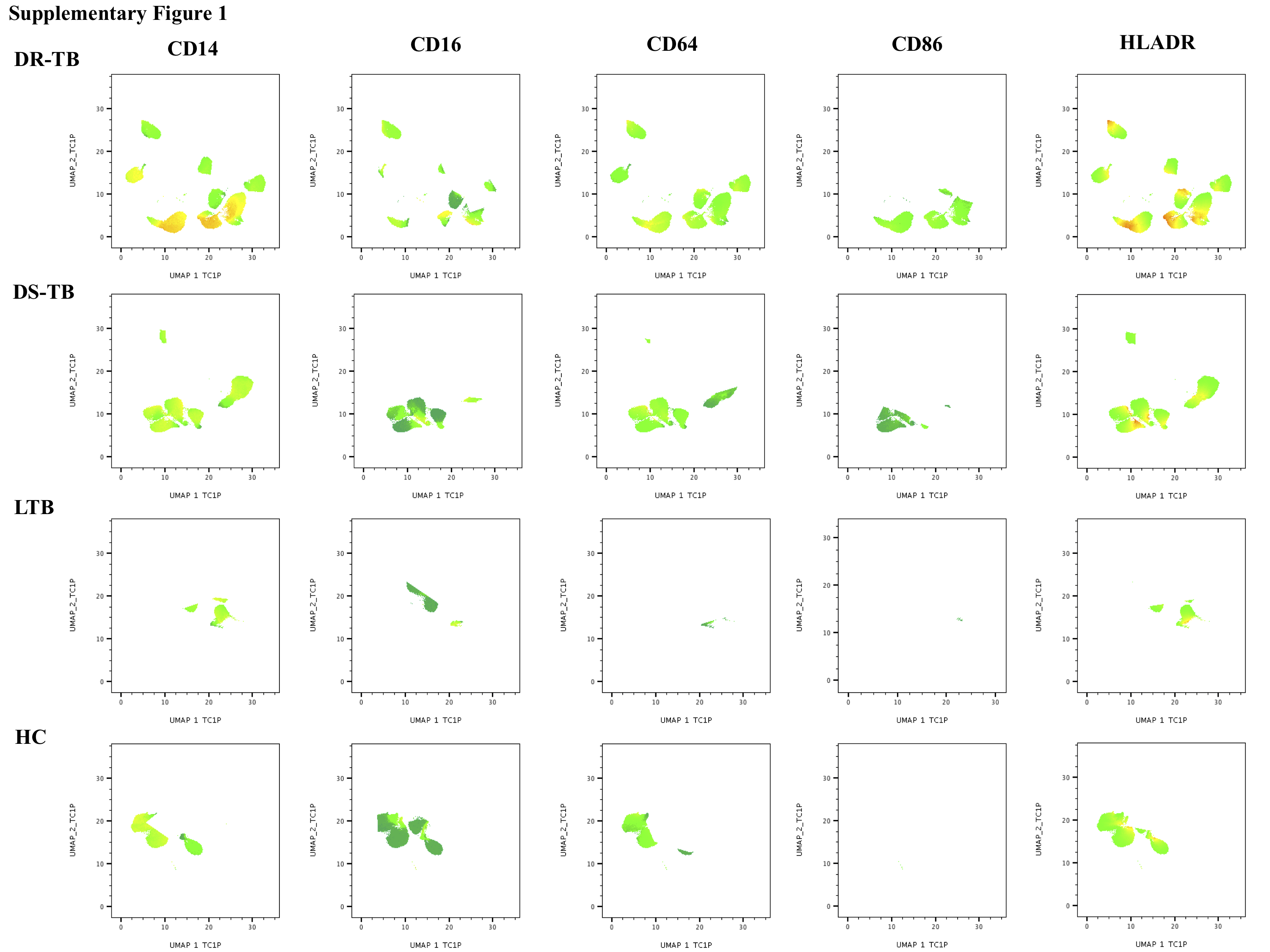

Supplement: Supplementary Figure 1 — Individual expression of monocyte (CD14. CD16, HLADR, CD64 and CD86) markers on UMAP between the study populations. [file Image_1.tiff]

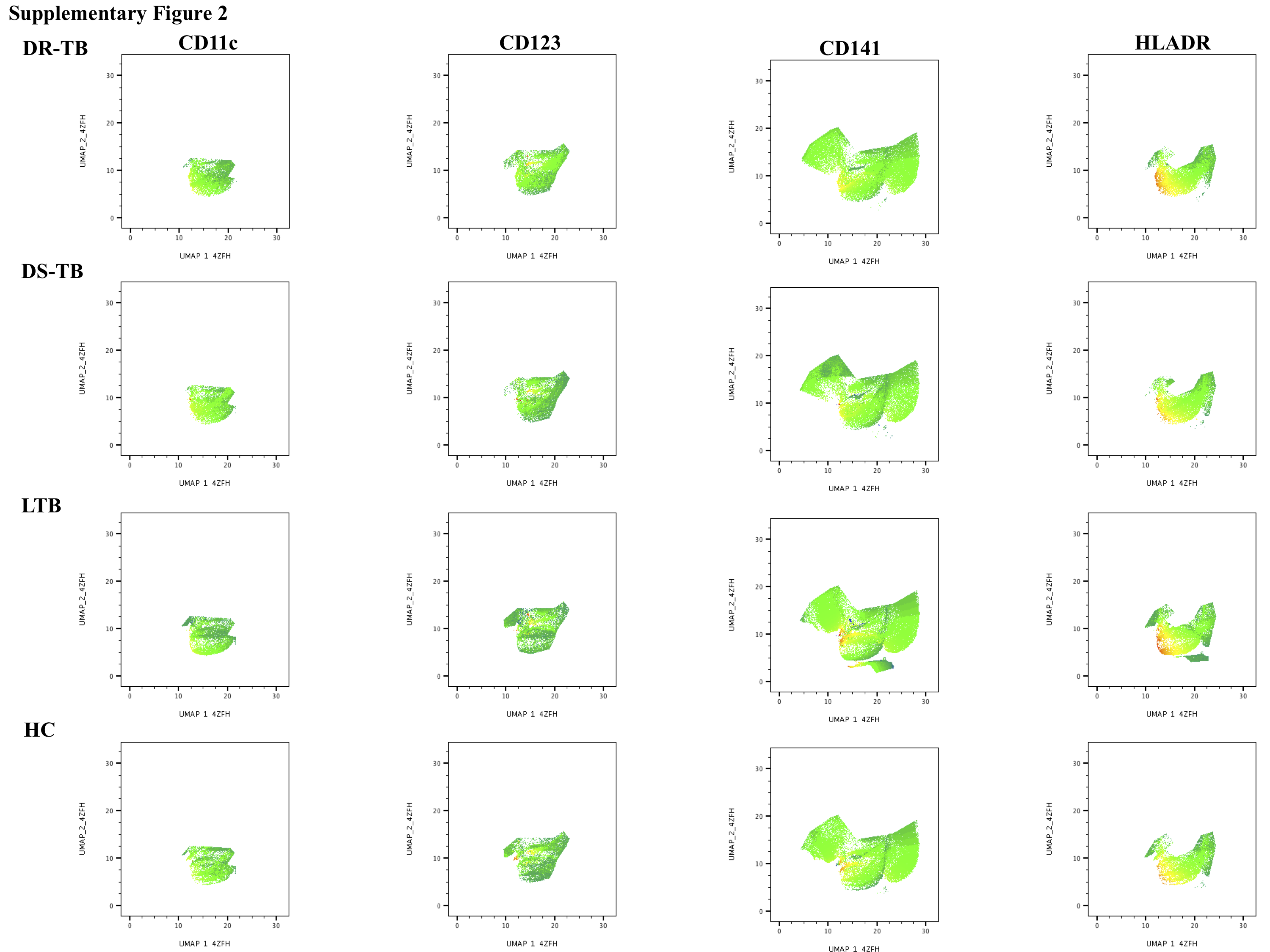

Supplement: Supplementary Figure 2 — Individual expression of DC (CD11c, CD123, CD141 and HLADR) markers on UMAP between the study populations. [file Image_2.tiff]
